# P-1341. Differential Frequency of Persister cells in Clinically Derived *Pseudomonas aeruginosa* Isolates after Exposure to Cefiderocol (FDC) or Ceftolozane/tazobactam (C/T)

**DOI:** 10.1093/ofid/ofae631.1518

**Published:** 2025-01-29

**Authors:** Aliaa Fouad, Samantha Nicolau, Pranita Tamma, Patricia J Simner, David P Nicolau, Christian M Gill

**Affiliations:** Hartford Hospital, Farmington, Connecticut; Sanofi, Boston, Massachusetts; Johns Hopkins School of Medicine, Baltimore, MD; Johns Hopkins School of Medicine, Baltimore, MD; Hartford Hospital, Farmington, Connecticut; Hartford Hospital, Farmington, Connecticut

## Abstract

**Background:**

Bacterial persistence is a phenomenon where a subpopulation of bacteria can survive high concentrations of an active antibiotic in the absence of genotypic alterations. Persisters are associated with chronic and recurrent infections for pathogens including *Pseudomonas aeruginosa*. Evaluating persister profiles of FDC and C/T against *P. aeruginosa* is warranted as they are needed for difficult-to-treat infections.

**Methods:**

Persisters were assessed using *in vitro* assays against 9 clinical *P. aeruginosa* isolates that developed resistance to C/T during therapy. Only index isolates (FDC and C/T-susceptible) were tested. Quantitative persister assays were performed using a stationary phase bacteria challenged with 10-fold MIC drug concentrations. Persisters were quantitated as the percent persisters at 24h and the log ratio (LR) difference in area under the curve for CFU (AUCFU) for each antibiotic alone referenced to control. Tolerance disk test (TDtest) was used to qualitatively detect persisters using full, half and quarter disks. Colonies within the inhibition zone after replacement of drug disk with a glucose disk indicated the presence of persister cells.

**Results:**

Percent persisters at 24h ranged from 4.1% to 64.3% and 1.9% to 81.1% for FDC and C/T, respectively. Percent persisters at 24h was lower for FDC compared with C/T groups in 6 of the 9 tested isolates. 8 of the 9 isolates had higher reduction in LR for FDC groups (range, -0.6 to -0.9), indicating an overall higher and more rapid bacterial reduction in FDC groups compared to C/T (range, -0.3 to -0.9). For FDC, 5/9 tested isolates lacked regrowth after replacement with glucose disk suggesting no persistence per the TDtest. For C/T, only 3 isolates lacked persister formation. TDtest results were consistent across disk sizes and concordant with the quantitative assay.

**Conclusion:**

These data link isolates that developed resistance to C/T with persister formation. FDC resulted in less bacterial persistence relative to C/T against 9 *P. aeruginosa* isolates. FDC’s siderophore mechanism may be advantageous over typical β-lactams via enhanced anti-persister activity. Clinical correlation of these findings are warranted as persisters can lead to antibiotic resistance and treatment failure.

**Disclosures:**

**Samantha Nicolau, PhD**, Sanofi Pasteur: Full time research scientist **Patricia J. Simner, PhD**, Affinity Biosensors: Grant/Research Support|BD Diagnostics: Grant/Research Support|bioMérieux: Grant/Research Support|Entasis: Personal fees|GeneCapture: Personal Fees|Merck: Personal fees|OpGen Inc: Grant/Research Support|Qiagen: Grant/Research Support|Shionogi: Personal fees|T2 Biosystems: Grant/Research Support **David P. Nicolau, PharmD**, CARB-X: Grant/Research Support|Innoviva: Grant/Research Support|Innoviva: Honoraria|Merck: Advisor/Consultant|Merck: Grant/Research Support|Merck: Honoraria|Pfizer: Advisor/Consultant|Pfizer: Grant/Research Support|Pfizer: Honoraria|Shionogi: Advisor/Consultant|Shionogi: Grant/Research Support|Shionogi: Honoraria|Venatorx: Grant/Research Support **Christian M. Gill, PharmD**, Cepheid: Grant/Research Support|Entasis: Grant/Research Support|Everest Medicines: Grant/Research Support|Shionogi: Grant/Research Support

